# Supplementary material for: Polymer architecture dictates multiple relaxation processes in soft networks with two orthogonal dynamic bonds
Source: Nat Commun. 2023 Nov 9;14:7244. doi: 10.1038/s41467-023-43073-w (PMC10636115; doi:10.1038/s41467-023-43073-w)
Supplement: Supplementary file 1 — Supplementary Information [file 41467_2023_43073_MOESM1_ESM.pdf]

Supplementary Information for

Polymer Architecture Dictates Multiple Relaxation Processes in Soft Networks with Two Orthogonal Dynamic Bonds

Sirui Ge[1,2], Yu-Hsuan Tsao[1,2] and Christopher M. Evans[1,2,3]*

[1]Department of Materials Science and Engineering, University of Illinois Urbana Champaign

[2]Materials Research Laboratory, University of Illinois Urbana Champaign

[3]Beckman Institute, University of Illinois Urbana Champaign

1. Synthesis of BOC-protected PDMS and GPC trace

To synthesize Boc-protected PDMS, the pendant PDMS precursor was dissolved in DCM, and excess di-tert-butyl dicarbonate (BOC anhydride) dissolved in DCM was added dropwise into the solution of PDMS precursors. After 30 hours of stirring at room temperature, The DCM was removed through rotary evaporation (Supplementary Fig. 1a). Then chloroform was added, and the product was dialyzed against chloroform to remove the excess di-tert-butyl dicarbonate. After that, the chloroform was removed through rotary evaporation, and the product was dried in vacuum oven overnight to remove the residual chloroform. A typical 1H-NMR result is shown in Supplementary Fig. 1b, indicating the successful Boc-protection of amine groups along the PDMS backbone. To synthesize telechelic Boc-protected pendant PDMS, the procedure is identical to that for pendant PDMS.

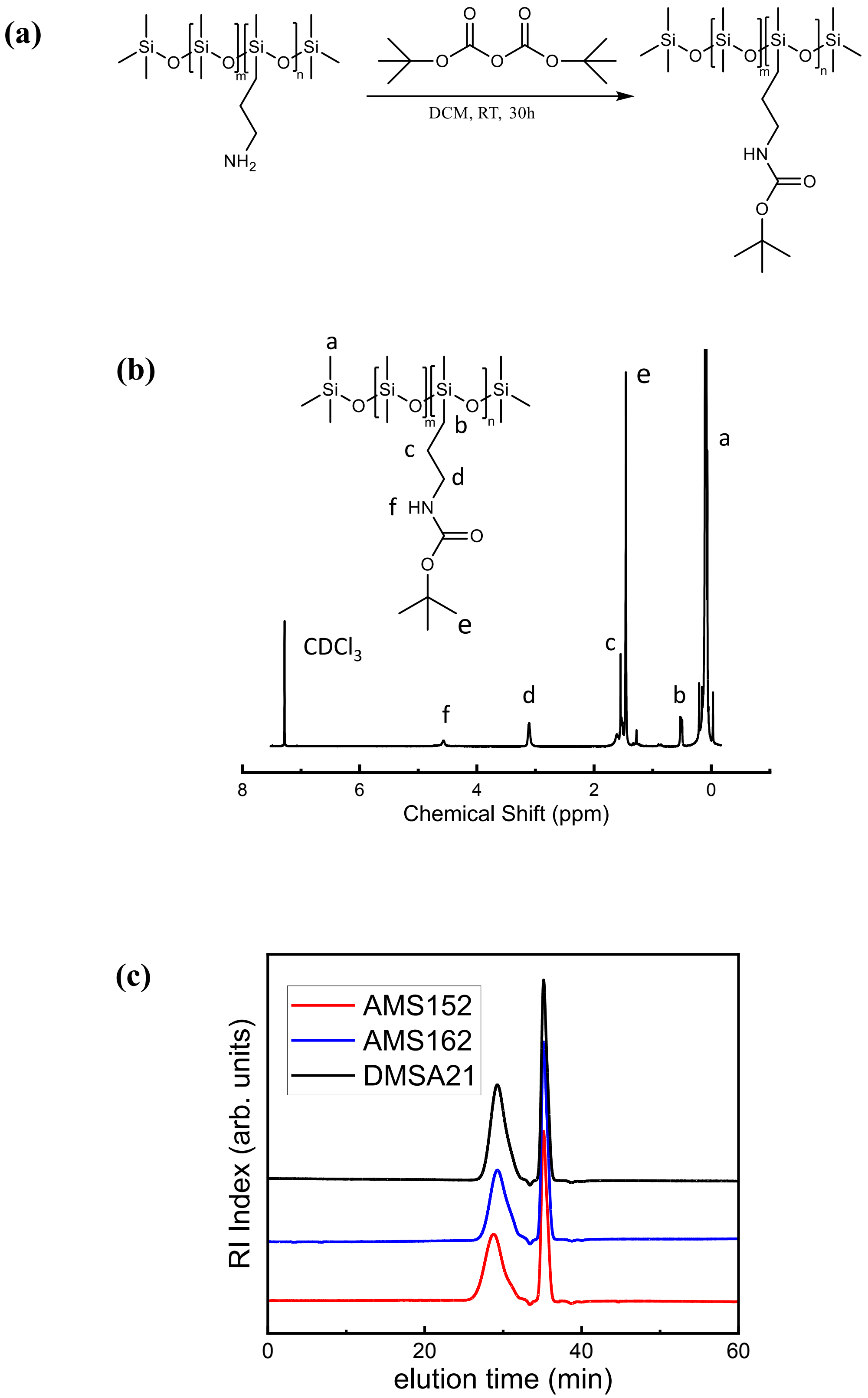

(a)
Si
O
Si
O
Si
O
Si
m
n
NH2
O
O
O
O
O
DCM, RT, 30h
HN
O
O
(b)
a
b
c
d
f
HN
O
O
e
CDCl3
e
a
c
b
d
f
8
6
4
2
0
Chemical Shift (ppm)
(c)
AMS152
AMS162
DMSA21
RI Index (arb. units)
0
20
40
60
elution time (min)

Supplementary Figure 1 (a) Synthesis route of pendant BOC-protected PDMS. (b) ^{1}H-NMR result for pendant Boc-protected PDMS. The peaks indicate the chemical shift of different hydrogens as is shown on the chemical structure. (c) GPC trace for all pendant (Pend-3.5: red; Pend-5.8: blue) and telechelic (black) PDMS precursors after treated by BOC anhydride. The peak at the short elution time is the sample peak, whereas the sharp peak at the long elution time is the solvent peak (Chloroform).

2. Glass transition temperature for all pendant and telechelic networks

Supplementary Table 1 Glass transition temperature for all pendant and telechelic networks

Sample	T_g (°C)
Tele-0/100	-123.9
Tele-25/75	-123.8
Tele-50/50	-122.8
Tele-75/25	-123.0
Tele-100/0	-122.9
Pend-3.5-0/100	-121.9
Pend-3.5-25/75	-121.01
Pend-3.5-50/50	-120.01
Pend-3.5-100/0	-119.73
Pend-5.8-0/100	-116.8
Pend-5.8-25/75	-115.82
Pend-5.8-50/50	-115.15
Pend-5.8-100/0	-115.14

3. Synthesis of telechelic PDMS-UPy with urea group and X-ray Scattering result

To synthesize telechelic PDMS-UPy with a urea group, the 2(6-isocyanatohexylaminocarbonylamino)-6-methyl-4[1H]pyrimidinone (UPy-HDI) was synthesized firstly following the previously reported method[1]. 2-amino-4-hydroxy-6-methylpyrimidine (1.1159g), 1,6-hexanediisocyanate (10.5g) and pyridine (0.7g) were mixed in a round bottom flask with a reflux condenser and stirred for 16 hours at 100 °C under nitrogen (Supplementary Fig. 2b). After the reaction, the sample was cooled down to room temperature before pentane was poured into the sample causing precipitation. Filtration was used to remove the solvent and the product was further washed by acetone three times. Then the sample was dried in a vacuum oven overnight at room temperature. The ^{1}H NMR result confirms the successful synthesis of product (Supplementary Fig. 2c). After UPy-HDI was successfully synthesized, excess UPy-HDI was dissolved in chloroform and then added into telechelic PDMS-NH_2 (1g) solution (chloroform as solvent). The reaction was conducted at room temperature for four hours under nitrogen (Supplementary Fig. 2d). After that, NH_2 functionalized silica gel was added in the solution and stirred at room temperature overnight before the silica gel was removed by filtration. Finally, chloroform was removed by rotary evaporation, and the sample was dried in vacuum oven at 333K overnight to acquire the product. The 1H NMR result confirms the successful synthesis of product (Supplementary Fig. 2e).

(a)

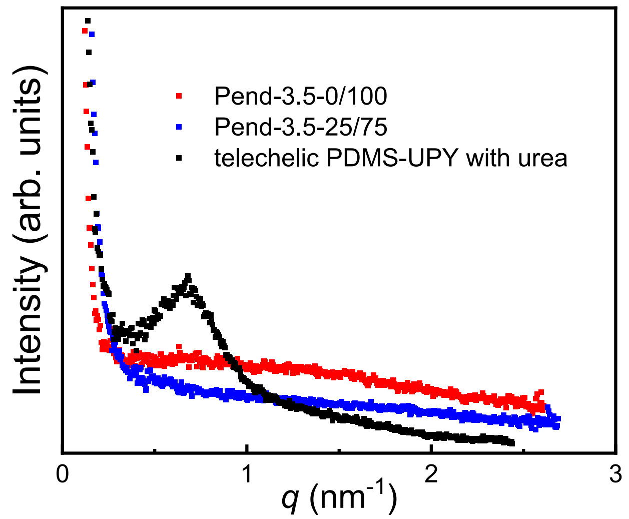
Pend-3.5-0/100
Pend-3.5-25/75
telechelic PDMS-UPY with urea
Intensity (arb. units)
0
1
2
3
q (nm-1)

(b)

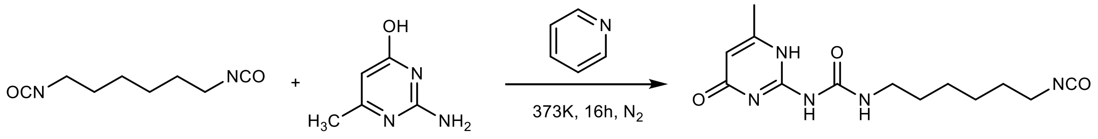
OCN
NCO
+
OH
N
H3C
N
NH2
N
373K, 16h, N2
NH
O
O
N
N
H
N
H
NCO

(c)

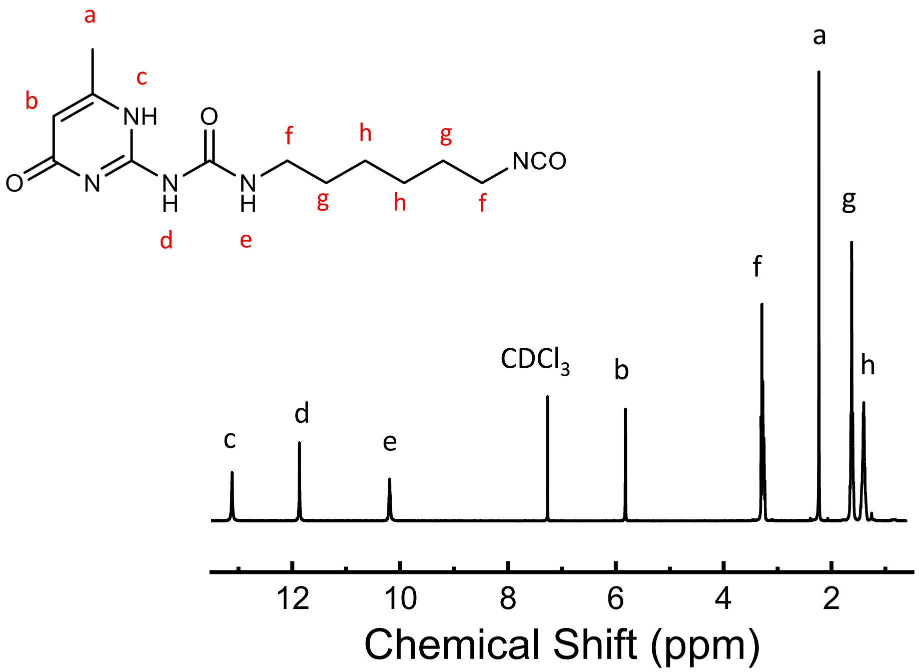
a
b
c
NH
O
f
h
g
NCO
O
N
N
H
N
H
g
h
f
d
e
a
g
f
h
CDCl3
b
c
d
e
12
10
8
6
4
2
Chemical Shift (ppm)

(d)

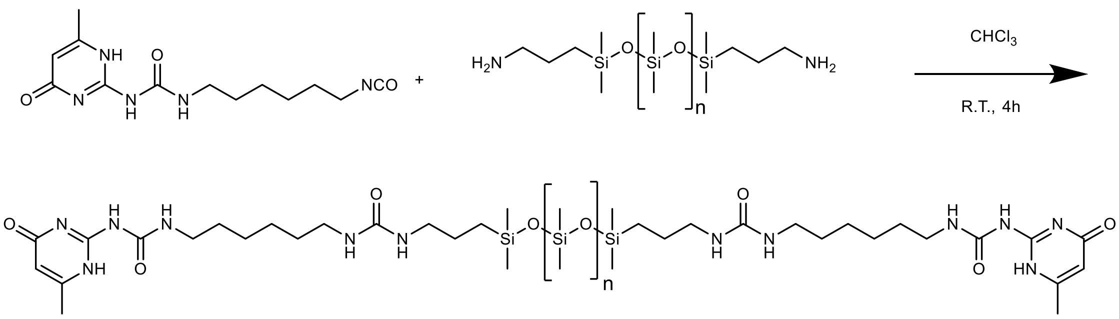

(e)

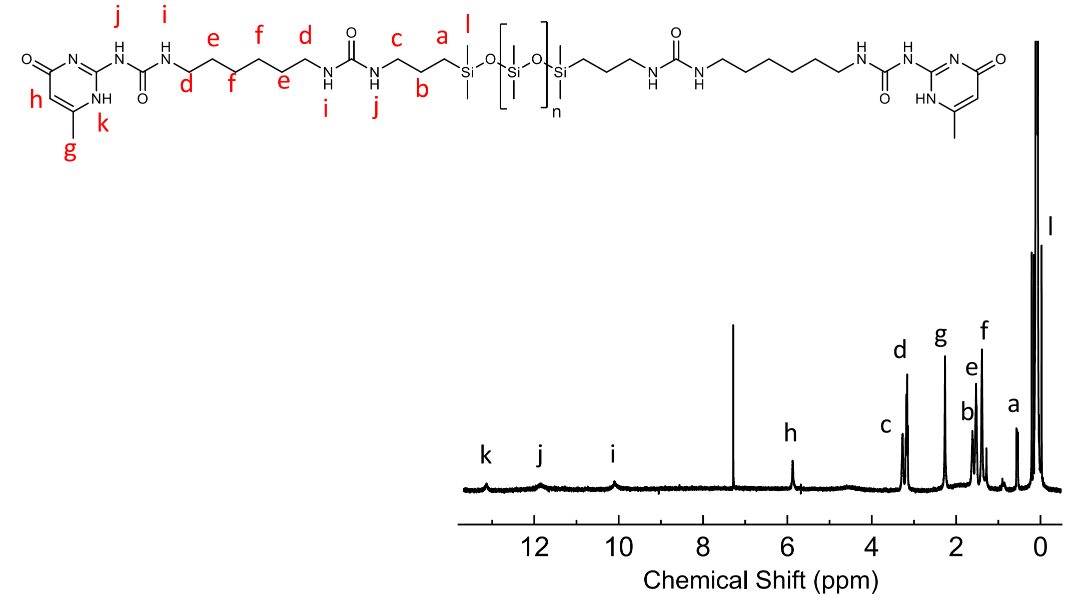

Supplementary Figure 2 (a) Small angle X-ray Scattering (SAXS) result of Pend-3.5-0/100 (red), Pend-3.5-25/75 (blue) and Tele-0/100 with urea (black). (b) Synthesis route of UPy-HDI building block. (c) ^{1}H-NMR result for UPy-HDI building block. The peaks indicate the chemical shift of different hydrogens as is shown on the chemical structure. (d) Synthesis route of telechelic-UPy with urea. (e) ^{1}H-NMR result for telechelic-UPy with urea. The peaks indicate the chemical shift of different hydrogens as is shown on the chemical structure.

4. **Comparison of Pendant PDMS with 50% of amine functionalized by imine crosslinker (No UPy functionalization and Pendant PDMS with mixed network (mixing ratio: 50/50)**

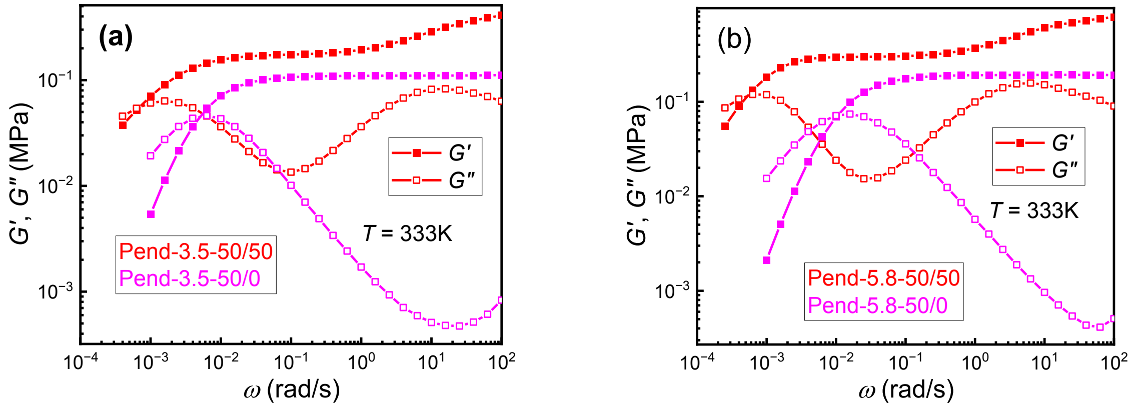

Supplementary Figure 3 Comparison of *G'(ω)* (closed symbol with solid line), *G''(ω)* (open symbol with solid line) spectra of (a) Pend-3.5-50/50 (red) and Pend-3.5-50/0 (pink), (b) Pend-5.8-50/50 (red) and Pend-5.8-50/0 (pink) measured at 333K.

5. Comparison of the shear modulus spectra of the Pendant PDMS with permanent crosslinker

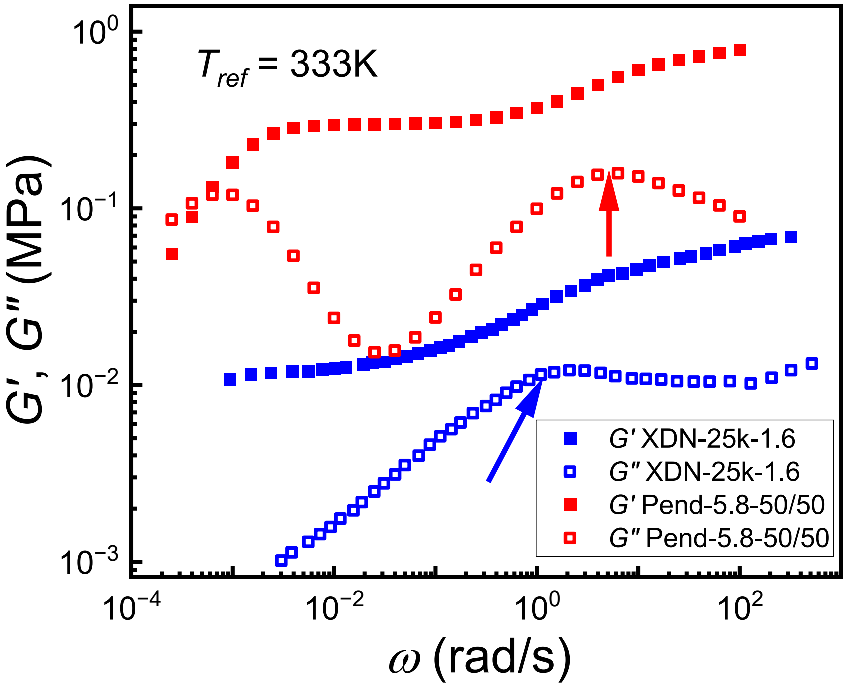

Supplementary Figure 4. Comparison of *G'(ω)* (closed symbol), *G''(ω)* (open symbol) spectra of the pendant PDMS mixed network (red) and the PDMS-UPy with permanent crosslinker (XDN-25k-1.6) (blue) (Data from Fig. 3b in Meng et al.[2])

6. Two adjacent relaxation modes for mixed network with pendant backbone

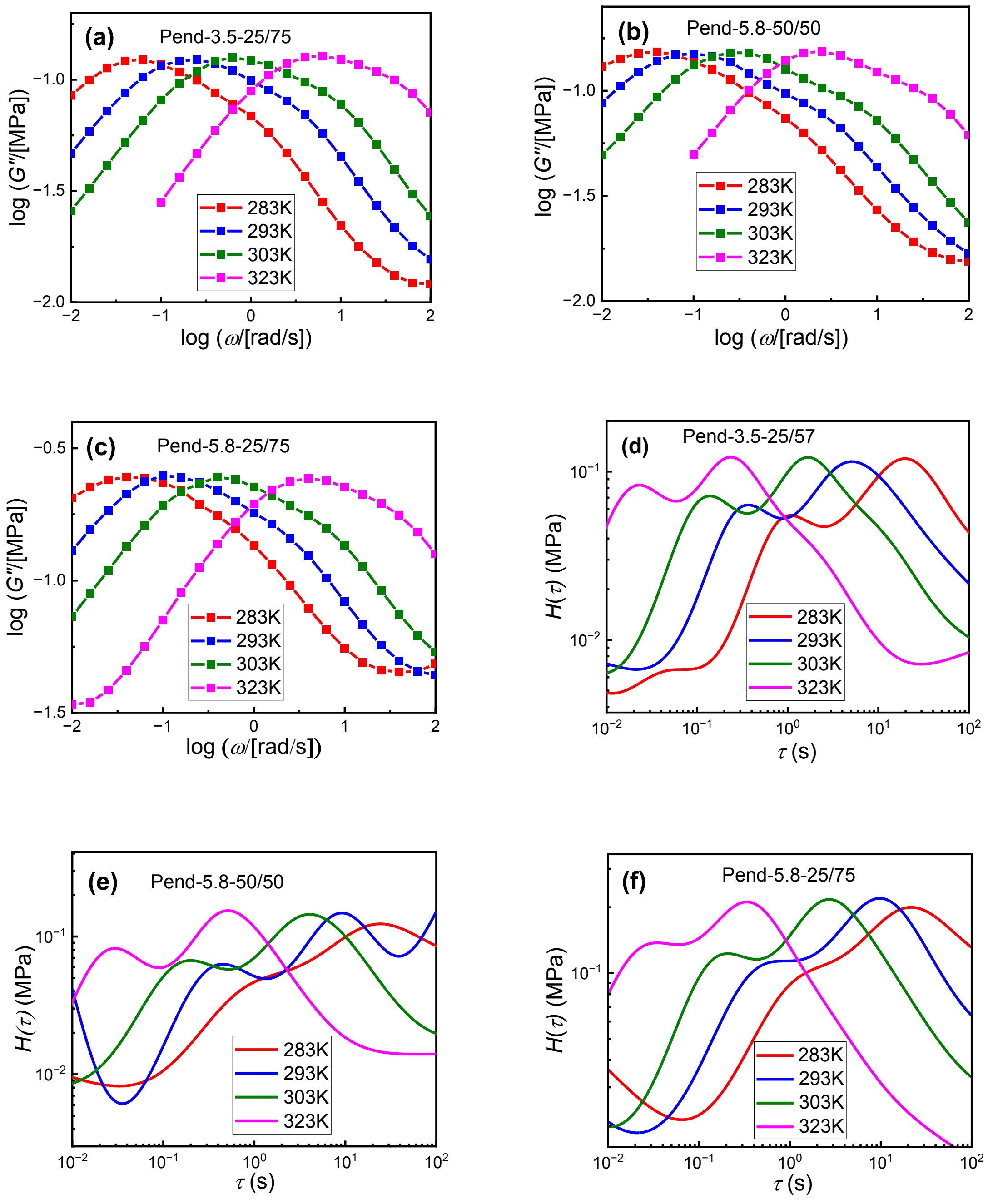

Supplementary Figure 5 (a) *G"(ω)* spectra of Pend-3.5-25/75 at different temperatures (different colors) in double logarithmic scale. (b) *G"(ω)* spectra of Pend-5.8-50/50 at different temperatures (different colors) in double logarithmic scale. (c) *G"(ω)* spectra of Pend-5.8-25/75 at different temperatures (different colors) in double logarithmic scale. (d) *H(τ)* spectra of Pend-3.5-25/75 at different temperatures

(different colors). (e) $H(\tau)$ spectra of Pend-5.8-50/50 at different temperatures (different colors). (f) $H(\tau)$ spectra of Pend-5.8-25/75 at different temperatures (different colors).

7. Two adjacent relaxation modes for UPy network with pendant backbone

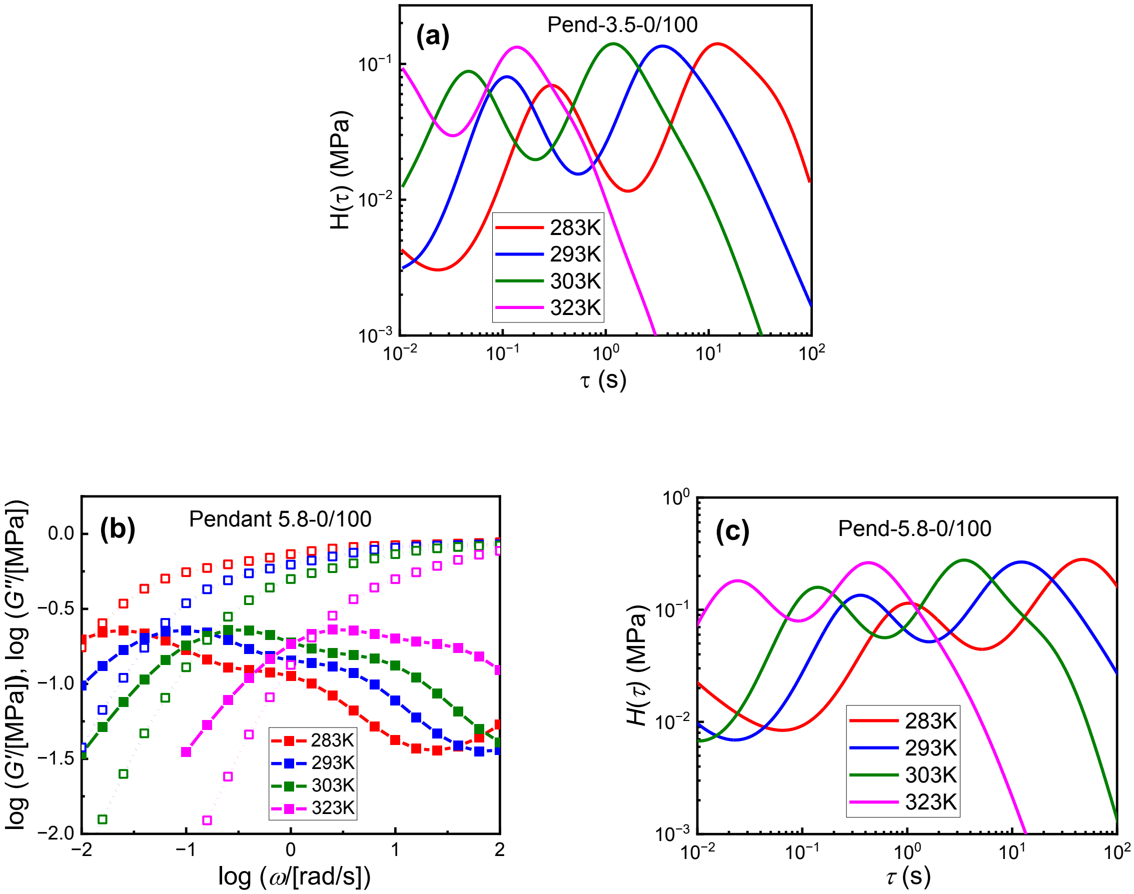

Supplementary Figure 6 (a) $H(\tau)$ spectra of Pend-3.5-0/100 at different temperatures (different colors). (b) $G'(\omega)$ (open symbol with dotted line), $G''(\omega)$ spectra of Pend-5.8-0/100 (closed symbol with solid line) at different temperatures (different colors) in double logarithmic scale. (c) $H(\tau)$ spectra of Pend-5.8-0/100 at different temperatures (different colors).

8. Dielectric M”(υ) spectra with the contribution of two relaxation processes

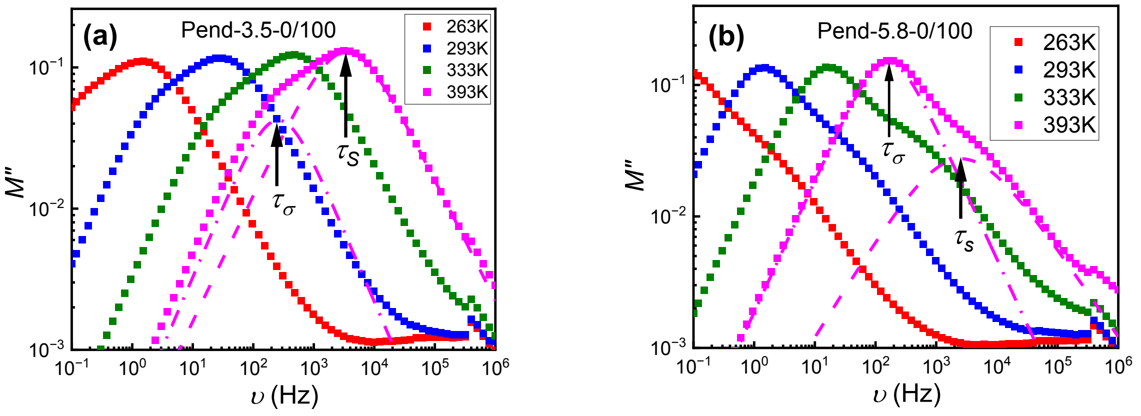

Supplementary Figure 7 Dielectric *M"(υ)* spectra of (a) Pend-3.5-0/100 and (b) Pend-5.8-0/100 at different temperatures (different colors). The position of τ_s and τ_σ on the spectra measured at 393K are labeled by arrows. The dashed lines indicate the contribution of bond dissociation processes. The dotdash line indicates the contribution of conductivity process.

9. Comparison of the timescale measured by rheology and dielectric spectroscopy for pendant PDMS with only UPy

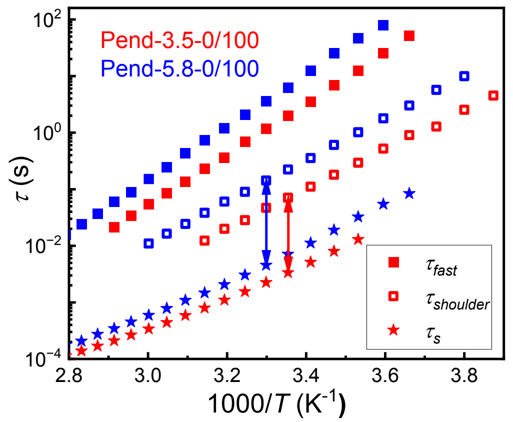

Supplementary Figure 8 Temperature dependance of τ_{fast} (closed square), $\tau_{shoulder}$ (open square) and τ_s (closed star) of Pend-3.5-0/100 (red) and Pend-5.8-0/100 (blue).

10. Two adjacent relaxation modes with telechelic backbone

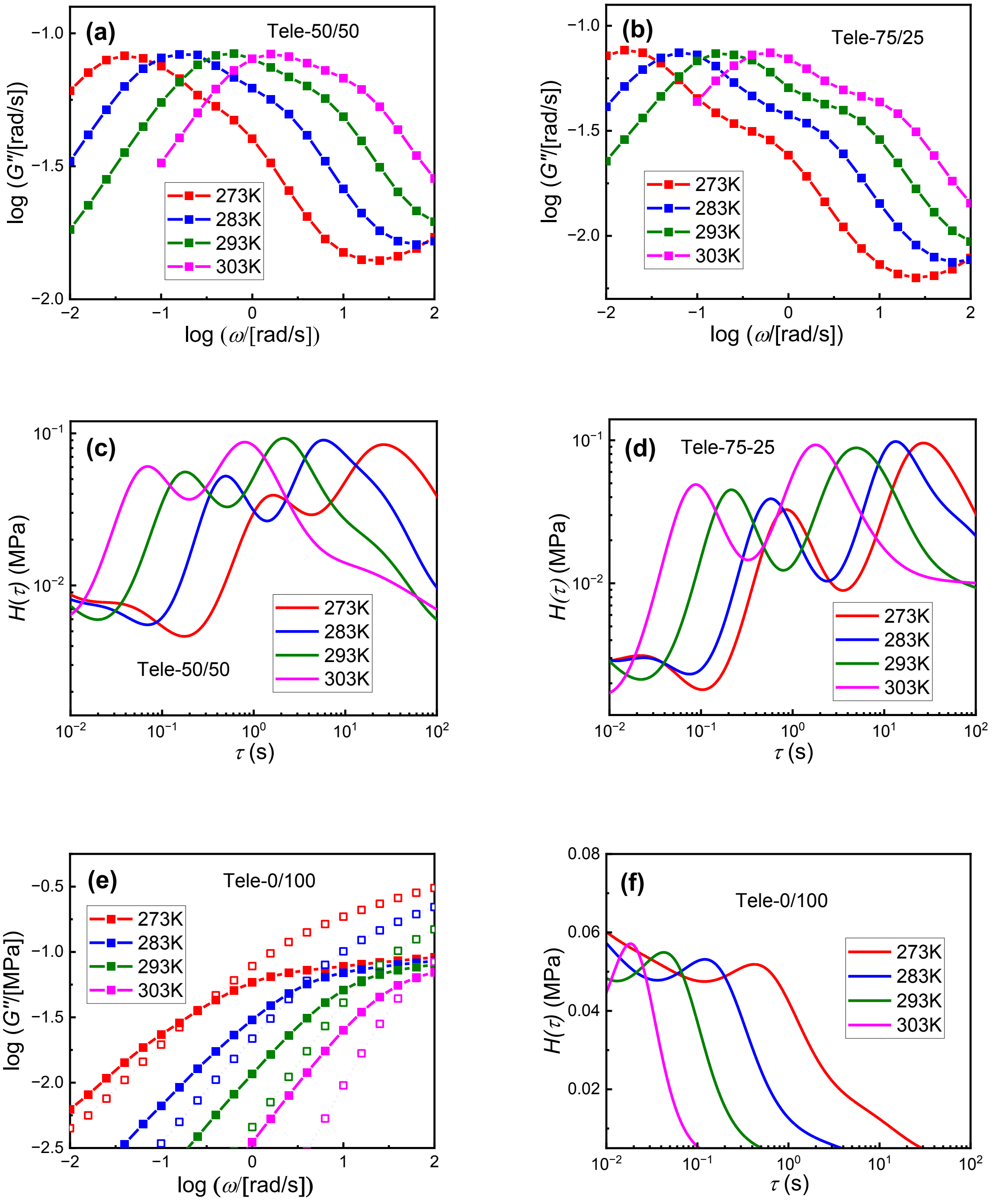

Supplementary Figure 9 (a) *G”(ω)* spectra of Tele-50/50 at different temperatures (different colors) in double logarithmic scale. (b) *G”(ω)* spectra of Tele-75/25 at different temperatures (different colors) in double logarithmic scale. (c) *H(τ)* spectra of Tele-50/50 at different temperatures (different colors). (d) *H(τ)* spectra of Tele-75/25 at different temperatures (different colors). (e) *G’(ω)* (open symbol with dotted lines), *G”(ω)* (closed symbol with solid lines) spectra of Tele-0/100 at different temperatures (different

colors) in double logarithmic scale. (f) $H(\tau)$ spectra of Tele-0/100 at different temperatures (different colors).

11. Synthesis of 2-(1-Imidazolylcarbonylamino)-6-methyl-4-[1H]-pyrimidinone (UPy-CDI)

The synthesis of 2-(1-Imidazolylcarbonylamino)-6-methyl-4-[1H]-pyrimidinone (UPY-CDI) follows a previously reported method[3]. Carbonyldiimidazole (CDI) (1.5g, 9mmol) and (2-Amino-4-hydroxy-6-methylpyrimidine) (0.75g, 6mmol) were mixed and dissolved in DMSO. Then the reaction was conducted in a round bottle flask with vigorous stirring for 24 hours under nitrogen at 333K. (Supplementary Fig. 10a). After that, the mixture was cooled to room temperature and the precipitate was collected through filtration with a glass frit and filter paper. The precipitate was also washed by acetone 3 times before being dried under vacuum at room temperature overnight. The final product was a white powder. Since UPY-CDI is not soluble in most common solvents[4], the IR spectra was used to characterize its structure. The result is shown in Supplementary Fig. 10b. The peak positions agree with the result reported in a previous publication[4].

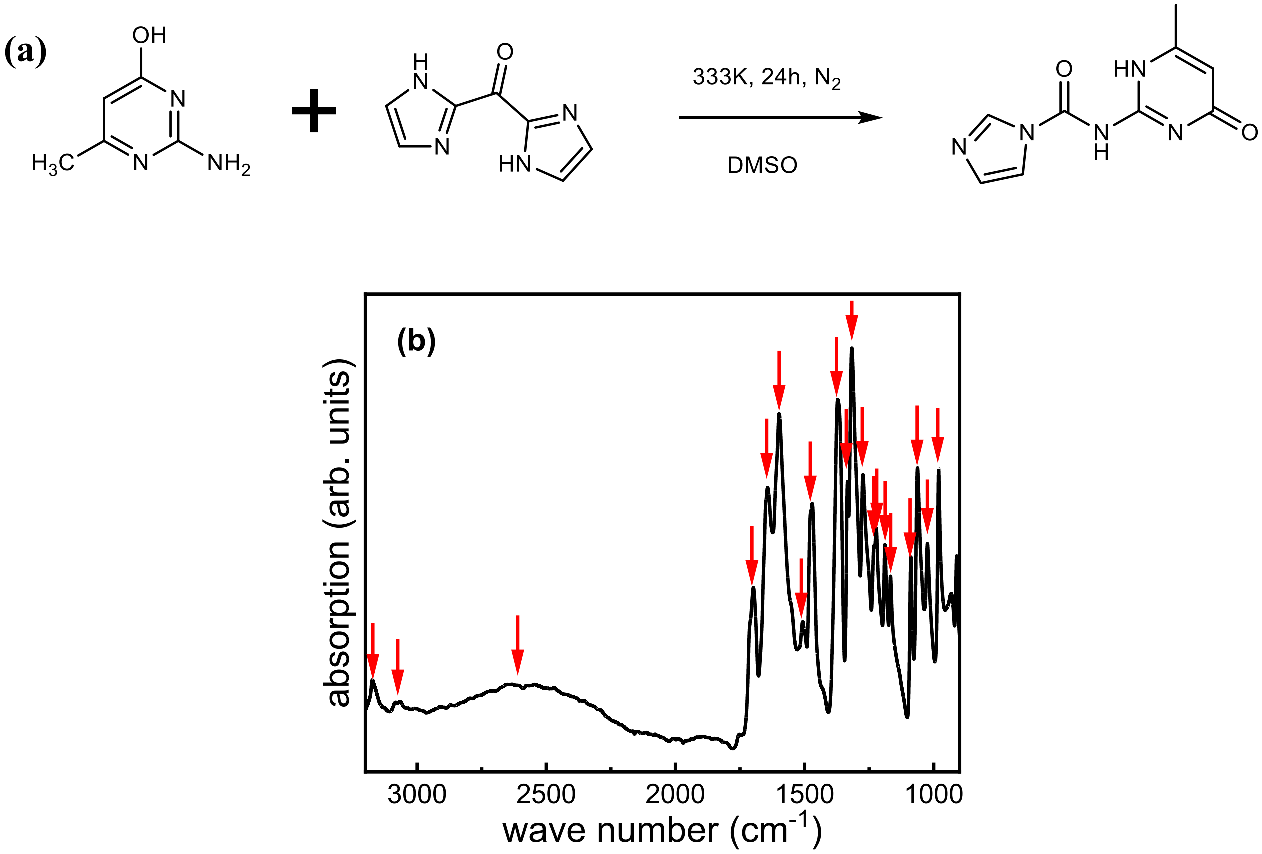

Supplementary Figure 10 (a) synthesis route of UPy-CDI building blocks. (b) IR result of UPy-CDI, red arrows indicate peak positions.

12. Synthesis of telechelic and pendant PDMS-UPy

(a)

(b)

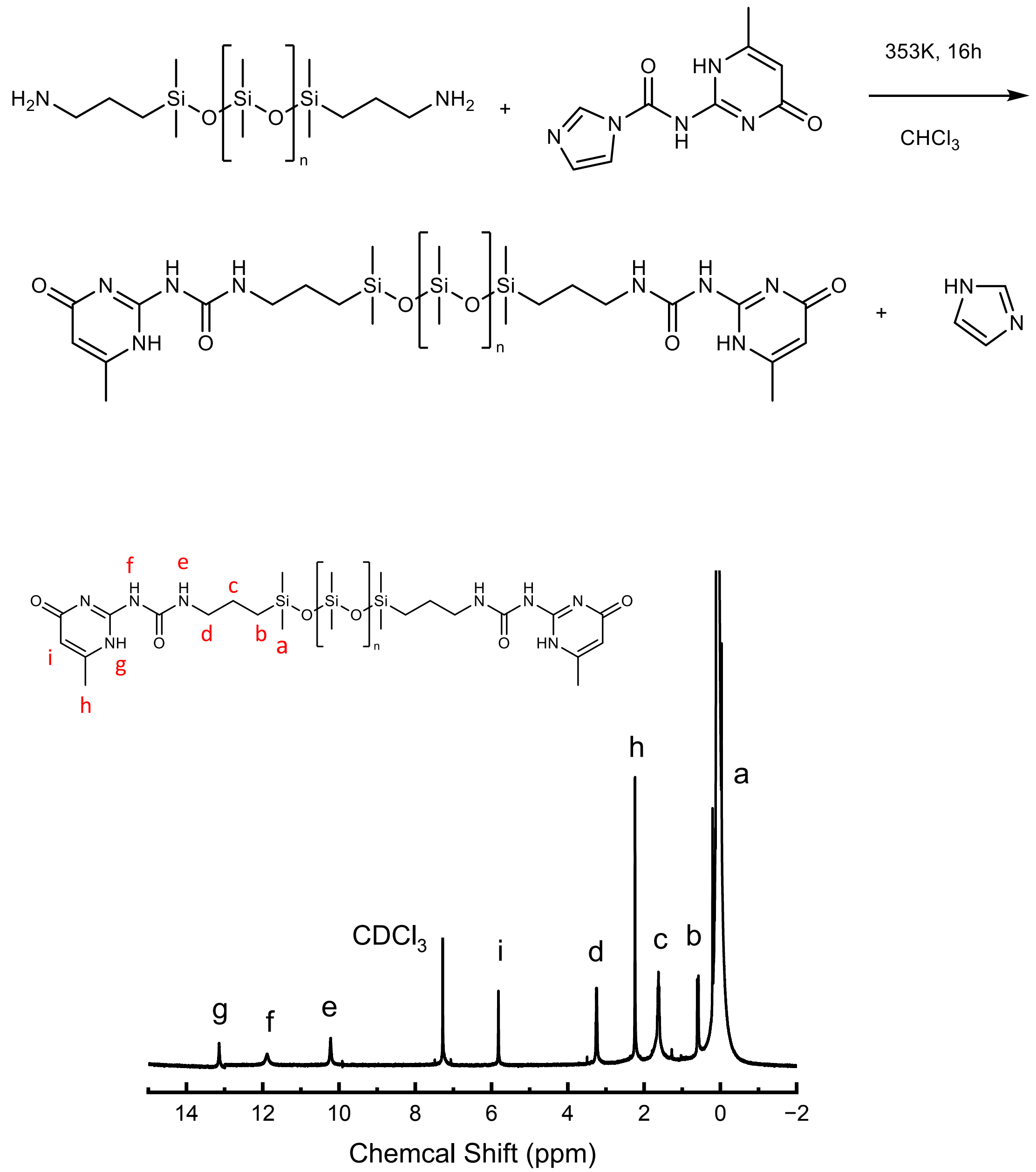

Supplementary Figure 11 (a) Synthesis route of telechelic PDMS-UPy. (b) ^{1}H-NMR result for the telechelic PDMS with UPy groups. The peaks indicate the chemical shift of different hydrogens as is shown on the chemical structure.

13. **Synthesis of telechelic and pendant PDMS with imine group**

(a)

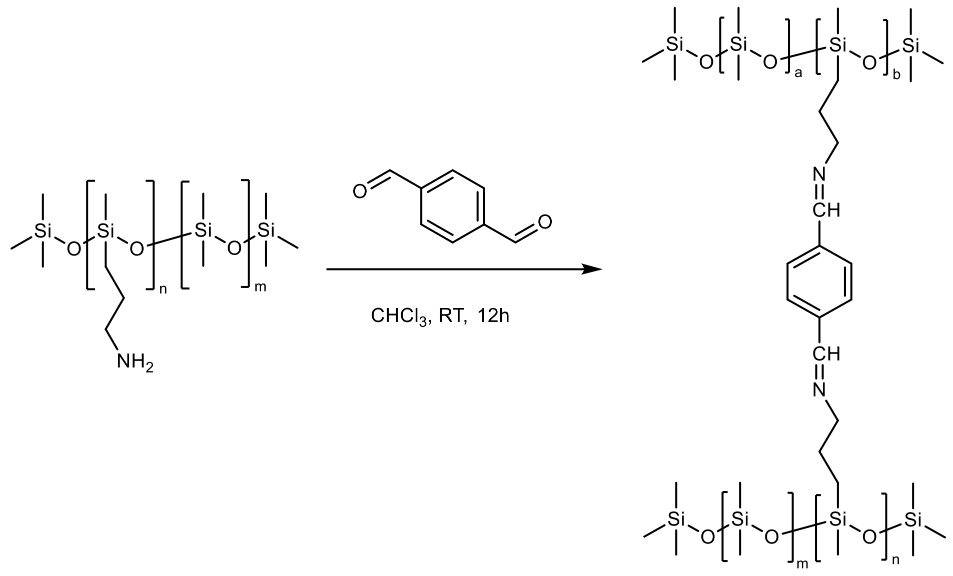

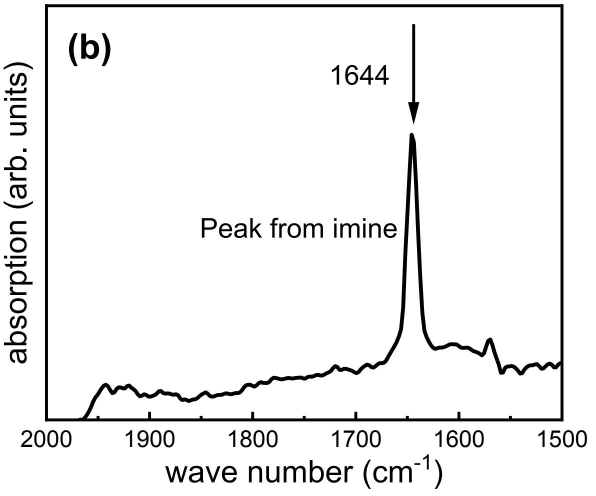

Supplementary Figure 12 (a) Synthesis route of pendant PDMS imine network. (b) IR spectra indicating imine group.

14. **NMR result of partially functionalized PDMS**

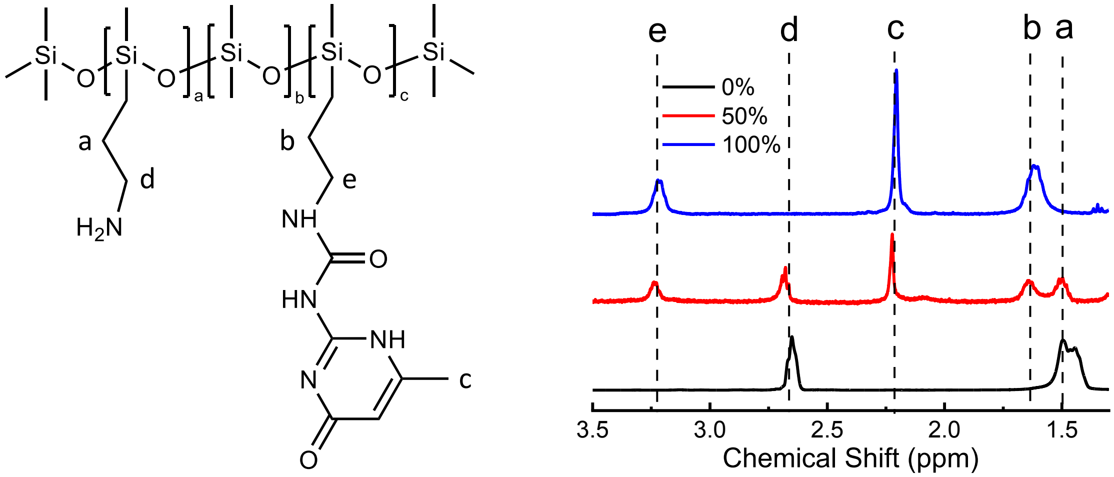

Supplementary Figure 13 ^{1}H-NMR result indicating pendant PDMS with 50% NH_2 functionalized by UPy.

15. Estimating the percolation threshold of imine network through Flory-Stockmeyer theory

To estimate the percolation threshold of imine network for the telechelic network, we applied the Flory-Stockmeyer theory:

The Flory-Stockmayer Theory predicts whether gelation (percolation) happens during step-growth polymerization, with reacting group A and B. Three assumptions are involved[5]:

(1) All functional groups on a branch unit have equal reactivity.
(2) All reactions happen between A and B
(3) No intramolecular reactions occur.

In addition, three types of monomer units are considered in the theory: linear units with two A-groups, linear units with two B-groups and linear units with two A-groups.

There are some important definitions in the theory:

f: is the functionality of the branch unit.

p_a is the probability that A has reacted.

p_b is the probability that B has reacted.

ρ is the ratio of number of A groups in the branch unit to the total number of A groups.

r is the ratio between total number of A and B groups, i.e, $r = \frac{p_B}{p_a}$.

The theory considers that the gelation occurs when $\alpha > \alpha_c$ in which

$$\alpha_c = \frac{1}{f-1} \quad \text{(S1)}$$

The α value can be presented as a function of either p_a or p_b, i.e.,

$$\alpha = \frac{r p_A^2 \rho}{1 - r p_A^2 (1-\rho)} = \frac{p_B^2 \rho}{1 - p_B^2 (1-\rho)} \quad \text{(S2)}$$

In our case of telechelic PDMS with imine, the imine crosslinker works as the branched unit with A group (aldehyde group) (with $f = 3$). The telechelic PDMS works as the linear unit with B group (amine group). As all the aldehyde group are on the imine crosslinker and no aldehyde was functionalized before reacting with telechelic PDMS, $\rho = 1$ and $p_a = 1$. Also, $r = p_B$. The value of p_B depends on the percentage of the amine group functionalized by UPy. In other words, p_B in our case indicates the percentage of amine finally reacted with imine crosslinker instead of reacting with UPy building block. Thus, by solving eq. S1 and eq. S2, we can easily get the critical point of getting percolation (gelation) is $p_B = 0.5$. In other words, at lease 50% of amine groups on the telechelic PDMS have to react with imine crosslinker. This agrees with our experimental result.

Supplementary References

1. Keizer HM, van Kessel R, Sijbesma RP, Meijer E. Scale-up of the synthesis of ureidopyrimidinone functionalized telechelic poly (ethylenebutylene). *Polymer* **44**, 5505-5511 (2003).

2. Meng Y, Xu W, Newman MR, Benoit DS, Anthamatten M. Thermoreversible siloxane networks: Soft biomaterials with widely tunable viscoelasticity. *Advanced Functional Materials* **29**, 1903721 (2019).

3. Bosman AW, Janssen HM, Van Gemert GML, Versteegen RM, Meijer EW, Sijbesma RP. Siloxane polymers with quadruple hydrogen bonding units Patent US7622131B2 (2009).

4. Teunissen AJ, Nieuwenhuizen MM, Rodríguez-Llansola F, Palmans AR, Meijer E. Mechanically induced gelation of a kinetically trapped supramolecular polymer. *Macromolecules* **47**, 8429-8436 (2014).

5. Flory PJ. Molecular size distribution in three dimensional polymers. I. Gelation1. *Journal of the american chemical society* **63**, 3083-3090 (1941).
